# SicknessMiner: a deep-learning-driven text-mining tool to abridge disease-disease associations

**DOI:** 10.1186/s12859-021-04397-w

**Published:** 2021-10-04

**Authors:** Nícia Rosário-Ferreira, Victor Guimarães, Vítor S. Costa, Irina S. Moreira

**Affiliations:** 1 CQC - Coimbra Chemistry Center, Chemistry Department, Faculty of Science and Technology, University of Coimbra, 3004-535 Coimbra, Portugal; 2 CNC - Center for Neuroscience and Cell Biology, University of Coimbra, Coimbra, Portugal; 3 Department of Sciences, University of Porto, Porto, Portugal; 4 INESC-TEC - Centre of Advanced Computing Systems, Porto, Portugal; 5 Department of Life Sciences, University of Coimbra, Calçada Martim de Freitas, 3000-456 Coimbra, Portugal; 6 CNC - Center for Neuroscience and Cell Biology, CIBB - Center for Innovative Biomedicine and Biotechnology, University of Coimbra, Coimbra, Portugal

**Keywords:** Disease-disease associations, Natural language processing, Biomedical text-mining, Deep learning, Blood cancers

## Abstract

**Background:**

Blood cancers (BCs) are responsible for over 720 K yearly deaths worldwide. Their prevalence and mortality-rate uphold the relevance of research related to BCs. Despite the availability of different resources establishing Disease-Disease Associations (DDAs), the knowledge is scattered and not accessible in a straightforward way to the scientific community. Here, we propose SicknessMiner, a biomedical Text-Mining (TM) approach towards the centralization of DDAs. Our methodology encompasses Named Entity Recognition (NER) and Named Entity Normalization (NEN) steps, and the DDAs retrieved were compared to the DisGeNET resource for qualitative and quantitative comparison.

**Results:**

We obtained the DDAs via co-mention using our SicknessMiner or gene- or variant-disease similarity on DisGeNET. SicknessMiner was able to retrieve around 92% of the DisGeNET results and nearly 15% of the SicknessMiner results were specific to our pipeline.

**Conclusions:**

SicknessMiner is a valuable tool to extract disease-disease relationship from RAW input corpus.

**Supplementary Information:**

The online version contains supplementary material available at 10.1186/s12859-021-04397-w.

## Background

Systems biology takes a holistic view on understanding biological systems, granting researchers an exceptional opportunity to dwell on previously scattered associations. Moreover, integrating knowledge from different sources on multiple diseases facilitates the understanding of Disease-Disease Associations (DDAs). The extraction of patterns from the evidence in relation to multiple diseases can ultimately establish networks encompassing such diseases. DDAs can be of various types and can be established considering different criteria as reviewed by Al-Eliwi and co-workers, all benefiting from network analysis approaches [1]. However, establishing DDAs using experimental data based on gene-disease associations can be tiresome, costly, and complex [2]. Hence, several strategies were proposed like: (i) Disease Ontology (DO), which integrates concepts from a plethora of sources to define related pathologies [3] or (ii) DisGeNET that defines DDAs as diseases that share, at least, a common gene or variant among the gene-disease associations considered in the database. DisGeNET comprises data retrieved from several approaches as expert curated resources, animal models, inferred data from Human Phenotype Ontology (HPO) and variants-related resources, and previously mentioned gene-disease associations [4].

In this work, our goal was to retrieve DDAs while lifting the strain on establishing or possessing prior gene-disease lists, and we concentrated herein on cancer with a particular focus on Blood Cancers (BCs). Cancer is a complex and multifactorial disease for which there is a widespread availability of related literature [5], useful for biomarker research [6], drug discovery [7], biological pathways detection [8], among others, highlighting this technique's positive contribution to a highly demanding field of study. The strength of mining the literature towards building disease networks was tapped into on multiple occasions as recently reviewed by Rodríguez-González [9]. In particular, BCs or hematologic cancers, which affect the production and function of blood cells encompassing the leukemia, lymphoma and multiple myeloma families, represent a large percentage of overall detected cancers (higher than 13%) and have a mortality-rate higher than 8% in the USA [10], with over 720 K deaths worldwide each year [11].

Biomedical text-mining, henceforth Text-Mining (TM), has already been used as a strategy to disclose analogous associations [12, 13]. TM allows the retrieval of informative data from latent data within biomedical literature, namely toward data-driven analysis [14, 15]. TM has greatly benefited from the takeoff of Artificial Intelligence (AI) algorithms that can be used for Natural Language Processing (NLP), an AI subfield dedicated to bridge the gap between human language and the language of computers. TM systems are most often built as pipelines that encompass a variable number of steps depending on the general goal of the research. Nonetheless, the initial steps of Named Entity Recognition (NER) and Named Entity Normalization (NEN) are generally needed. NER processes the input text into a set of entities, whilst NEN maps an entity into a concept in a terminology [16, 17]. State-Of-The-Art methods (SOTA), such as Bidirectional Encoder Representations from Transformers for Biomedical Text Mining (BioBERT), provide excellent results for the NER step of TM, attaining human-like performance [17]. However, there is still a lack of NEN models that can tackle multiple categories as well as a need for combined NER and NEN strategies to be time-smart and enhance accuracy by integrating other data types such as ontologies directly upon the entity’s retrieval step [14].

**Fig. 1:**
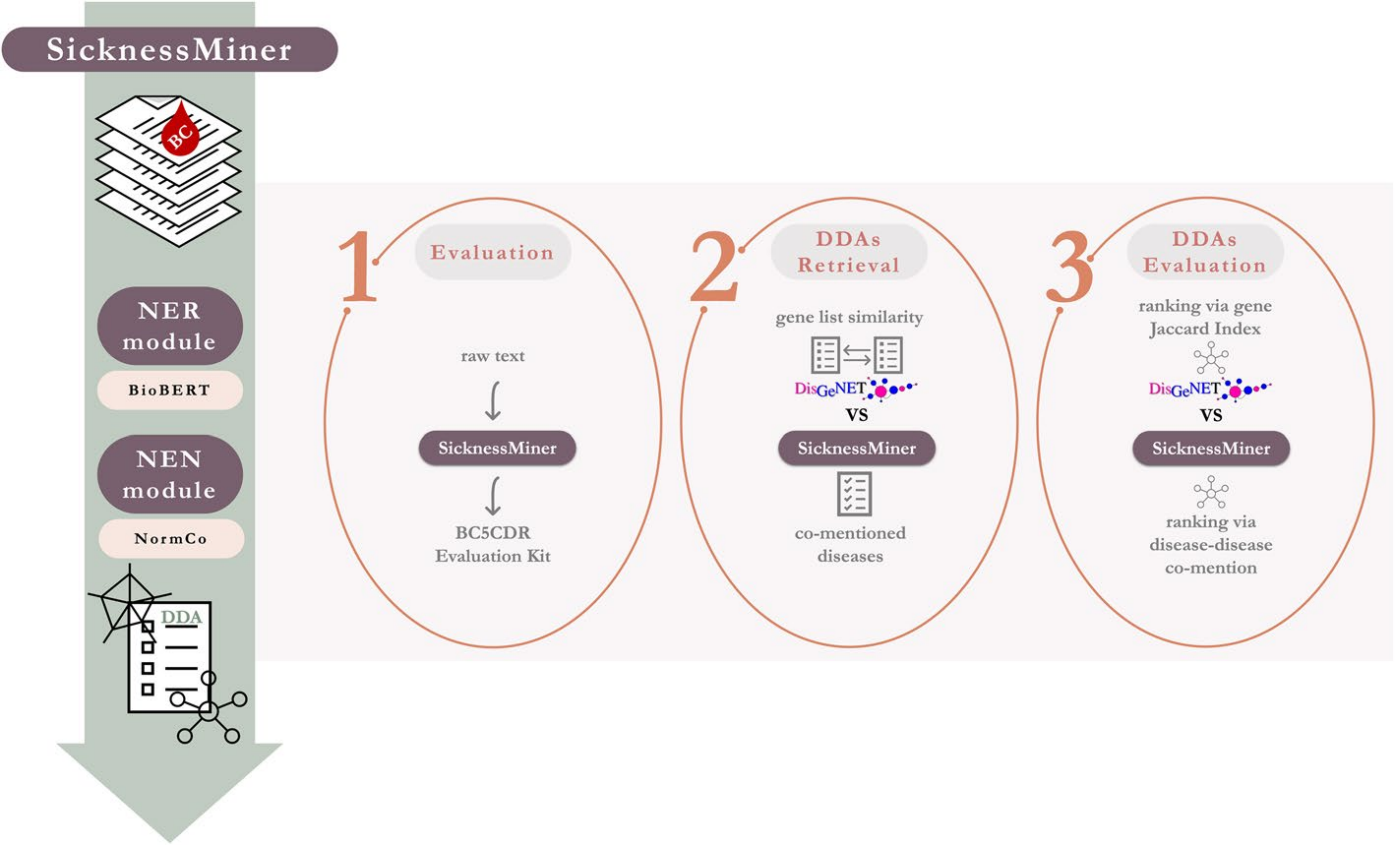
SicknessMiner pipeline: a TM approach for DDAs. SicknessMiner is a two-step pipeline integrating subsequent modules for NER and NEN. First, from the RAW text input, an entity's list is plotted according to co-mentions with more than 1 BC type. To evaluate SicknessMiner, we used the BC5CDR evaluation kit. Finally, DDAs were doubly assessed via SicknessMiner and DisGeNET and further evaluation was performed for a better understanding of key improvements obtained herein

Herein, we present SicknessMiner,^1^ a leading-edge TM pipeline encompassing both NER and NEN steps. The pipeline uses SOTA models as BioBERT for NER and NormCo for NEN. SicknessMiner does not require a specific input format and, as such, has a broad application and it is easy-to-use. SicknessMiner was fine-tuned for diseases and is able to return them as entities mapped to Medical Subject Headings (MeSH) terms with top performance. Both models of SicknessMiner were trained on the NCBI Disease dataset [18]. SicknessMiner demonstrates that our chosen DDA evaluation, ranking via disease-disease co-mention in a set of scientific publications, can be highly useful to find meaningful relations. This is a powerful finding, since this technique relies only on NER and NEN models. Such high accuracy models can be easily constructed, widening SicknessMiner application as it does not require the existence of other ontologies or knowledge bases regarding each specific domain of application. To further evaluate our DDAs results, we compared them with a well-known database, DisGeNET, which classifies DDAs based on the similarity of their gene- or variant-disease lists. Figure 1 illustrates the methodological pipeline followed to attain, validate and evaluate SicknessMiner.

## Results

SicknessMiner two modules, NER and NEN, were trained using a corpus of 793 PubMed abstracts with over 6.8 K disease mentions mapped to 790 unique concepts either linked to the MeSH or the Online Mendelian Inheritance in Man (OMIM) databases. The NER task consists of identifying entities of interest in a given corpus. For example, given a RAW text, the output of the NER module is a set of text spans where the entities can be found, in our particular case, known diseases. After the NER step, the retrieved text spans related to the entities serve as input to the NEN module, which maps each text span to an entry in a given ontology. If an entity is not found in the ontology, it is mapped as *unknown*. The main output of the SicknessMiner consists of a set of entities found in a given corpus, with their respective IDs corresponding to the key ontology, as well as the place in the text where the entities were found.

Both models of SicknessMiner were trained on the NCBI Disease dataset [18]. For the NER, we started the BioBERT model with the BioBERT v1.1 pre-trained weights [17] and fine-tuned them in the NCBI Disease train dataset using the default parameter of the system made available by Lee et al. [17]. For the NEN, we trained the NormCo [19] on the NCBI Disease train dataset using the default parameter of the system.

### SicknessMiner evaluation

We choose a simple but efficient method to DDA evaluation: ranking via disease-disease co-mention. To evaluate the performance of our method in the NCBI Disease test set, we used the evaluation kit^2^ from the BC5CDR task to compute the precision, recall, and F1-score for both the NER and NEN steps. The BC5CDR evaluation kit is a program that receives a set of labelled text from the user and, by using the predictions of the model as input, computes these three performance metrics as output in order to assess the efficiency of the model developed by the user. SicknessMiner attained a precision of 0.87, recall of 0.89 and F1-score of 0.88 for the NER module; and a precision of 0.80, recall of 0.83 and F1-score of 0.81 for the NEN module given a perfect NER. When the NER results were considered, the NEN module achieved a precision of 0.72, recall of 0.79 and F1-score of 0.76.

### DDAs retrieval

We applied DDA retrieval to BCs as already mentioned. We queried PubMed for “((leukemia[Title/Abstract]) OR (multiple myeloma[Title/Abstract])) OR (lymphoma[Title/Abstract])” and retrieved over 390 K titles and abstracts. We merged all the results and submitted them to the SicknessMiner pipeline. To evaluate the results obtained by SicknessMiner, we compared them against DisGeNET with the Concept IDs for the BCs types: C0023418, C0024299, and C0026764 for leukemia, lymphoma and multiple myeloma, respectively. DisGeNET was chosen for direct performance comparison as the existing alternative models exhibit a few difficulties as these sometimes are not publicly available, do not encompassed the same group of diseases, or can even involve a complex methodological approach, which goes against our main aim of attaining an easy-to-use integrative pipeline [13, 20–25]. Whilst DisGeNET retrieved a total of 57,624 co-mentions between 22,611 unique diseases and one of the diseases of interest (Leukemia, Lymphoma or Multiple Myeloma), SicknessMiner retrieved 12,263 co-mentions between 5443 unique diseases.

### DDAs evaluation

**Fig. 2:**
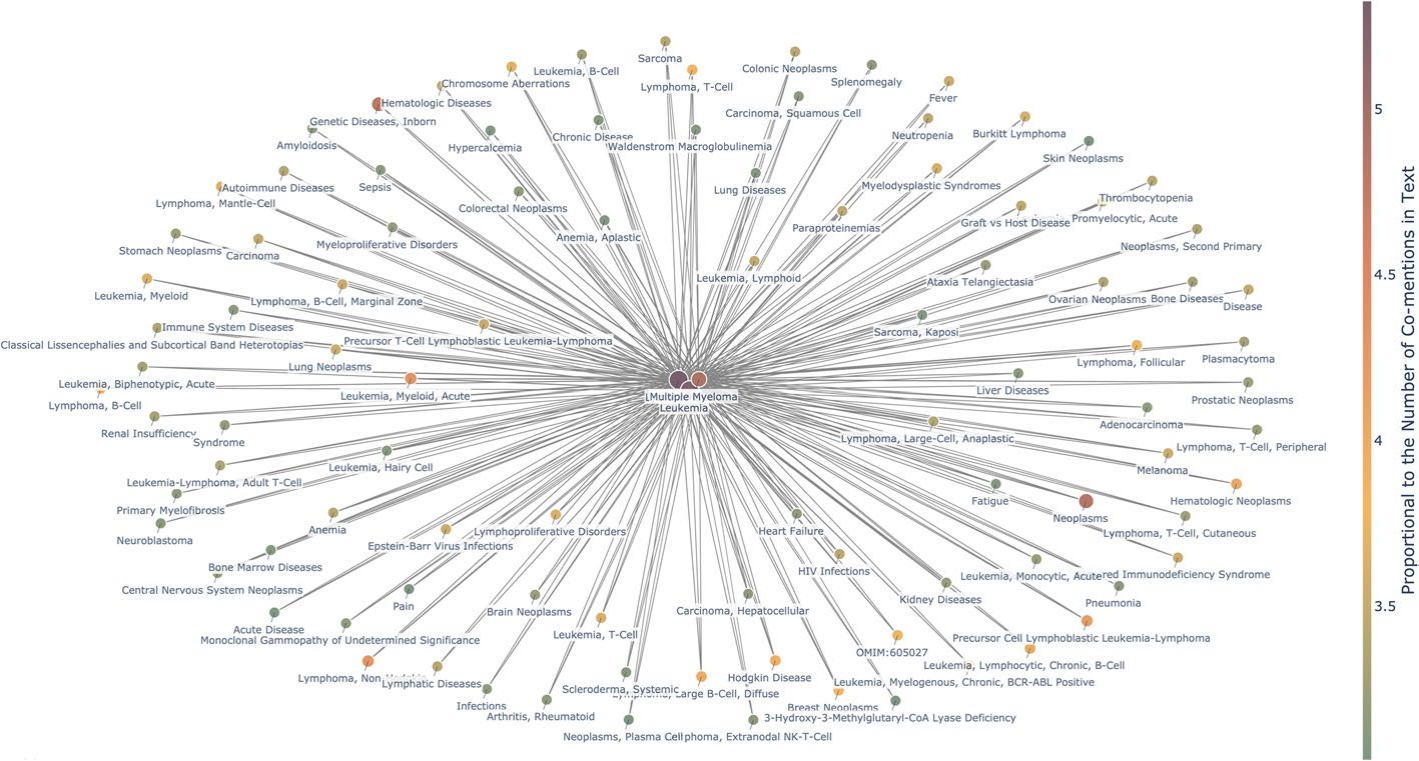
SicknessMiner Top 100 co-mentioned entries (in this case all entries are related to the 3 BCs since the query was combined)

**Fig. 3:**
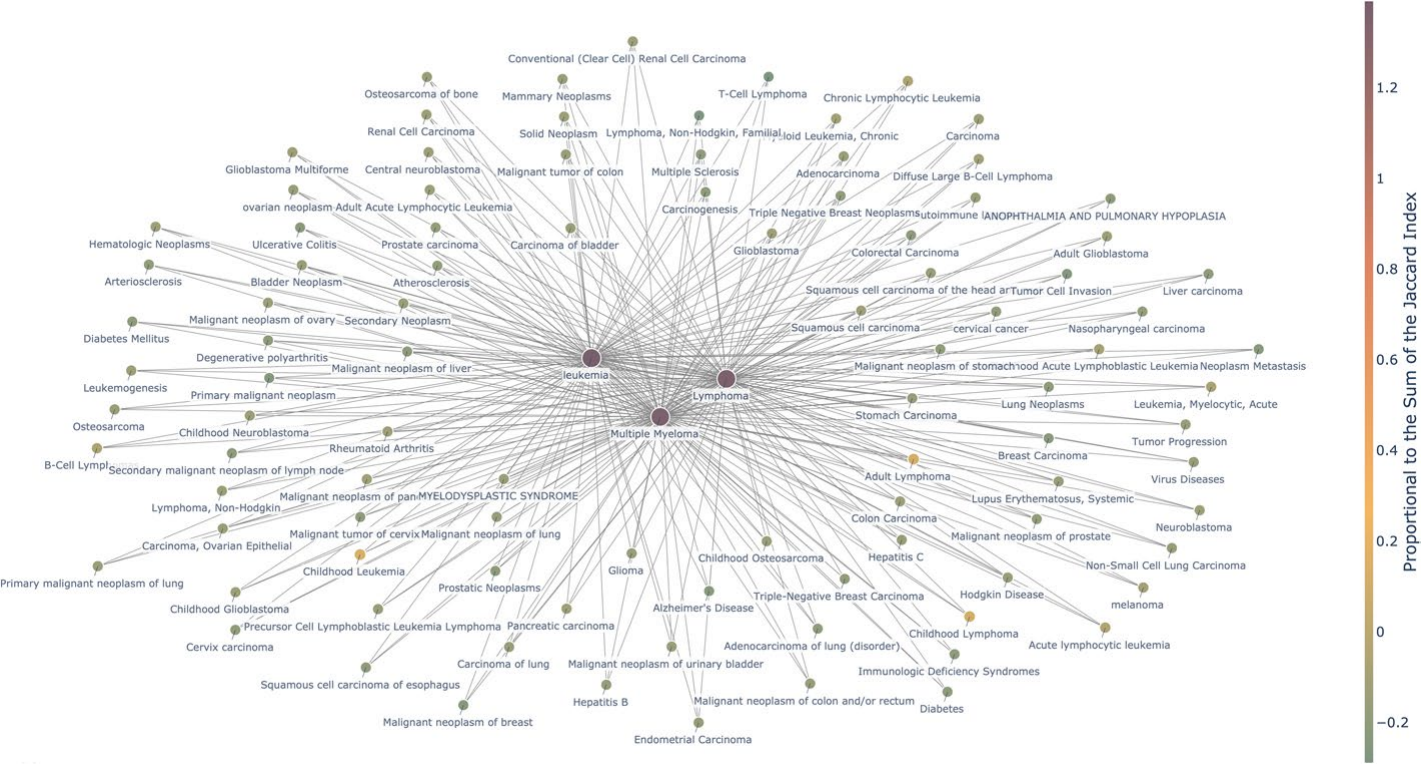
DisGeNET Top100 entries that are correlated with 2 or 3 BCs and share, at least, 20 genes or variants between diseases

**Fig. 4:**
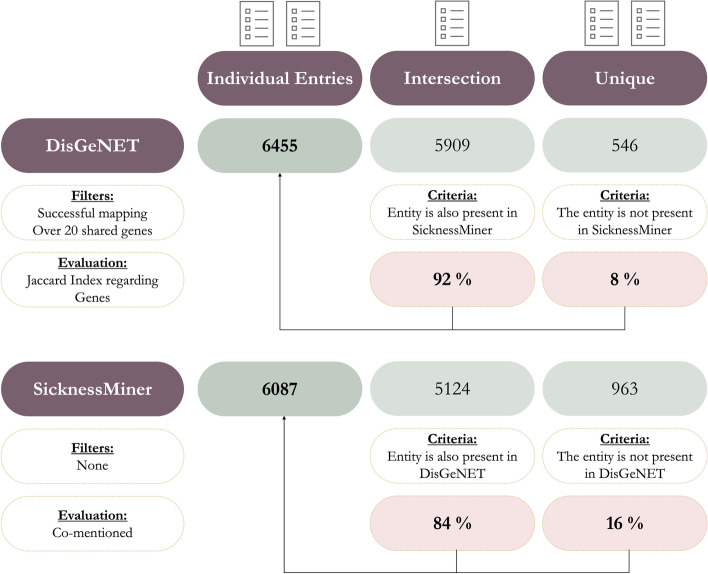
SicknessMiner and DisGeNET comparison

SicknessMiner was qualitatively and quantitatively compared against the results obtained through DisGeNET. To this end, we plotted a graph for each system, where each node in the graph corresponds to a disease and each edge connects two related diseases (Figs. 2 and 3; full results tables are available as Additional file 1). In Fig. 2, every resulting node in the graph is one of the top 100 hits with more shared genes connected to 2 or 3 BCs simultaneously from DisGeNET. In Fig. 3, each graph node represents one of the top 100 hits regarding the number of co-mentions to the 3 BCs via SicknessMiner. To make some sense of the totality of results and how the two systems compare, we also performed quantitative analysis for each approach (Fig. 4). To improve readability, we filtered the 100 most relevant nodes. For the DisGeNET, the process was straightforward, since it already provided the data related to our target diseases (Leukemia, Lymphoma or Multiple Myeloma). The set of genes of the target and related disease were sorted using the Jaccard index, and the top 100 diseases related to at least two target diseases were chosen. For SicknessMiner, we considered that two diseases were related if both of them appeared together in a paper title or abstract (i.e., they are co-mentioned). Afterwards, we ranked the co-mentions based on the number of times they were in the text set and chose the top 100 related to at least two target diseases. To attain realistic DDAs in DisGeNET, we considered a threshold for the similar genes of, at least, 20 shared genes to accept a DDA as positive. While SicknessMiner could retrieve overall minus 5% of DDAs (6455 vs 6087), 92% of the DDAs delivered by DisGeNET were also available in SicknessMiner. Also, SicknessMiner yielded close to 16% unidentified DDAs by DisGeNET, contrasting to only 8% diseases from DisGeNET that were unidentified in SicknessMiner (Fig. 4).

We also looked at the results from a biological angle since SicknessMiner’s goal was to retrieve diseases from biomedical literature, hence, it is domain-specific in its model. The majority of edges detected, thus the majority of the DDAs reported, are some type of cancer. However, in the top 100 edges, SicknessMiner retrieved a higher number of DDAs/edges representing non-cancerous diseases than DisGeNET, 22% and 4%, respectively. In terms of unique non-cancerous DDAs, SicknessMiner could find 17 entities against 3 in DisGeNET. The retrieved non-cancerous DDAs are listed in Table 1.

**Table 1:** Retrieved non-cancerous DDAs from SicknessMiner and DisGeNET

SicknessMiner				
Identifier	Related disease	Identifier	Blood cancer type	Co-mentions
C538324	3-Hydroxy-3-methylglutaryl-coa lyase deficiency	D008223	Lymphoma	907
D000163	Acquired immunodeficiency syndrome	D008223	Lymphoma	2358
D000163	Acquired immunodeficiency syndrome	D007938	Leukemia	906
D000686	Amyloidosis	D009101	Multiple Myeloma	1108
D000740	Anemia	D007938	Leukemia	992
D001327	Autoimmune diseases	D008223	Lymphoma	1680
D001847	Bone diseases	D009101	Multiple Myeloma	1548
D002869	Chromosome aberrations	D007938	Leukemia	2834
D002869	Chromosome aberrations	D008223	Lymphoma	1161
D007938	Classical lissencephaly and subcortical band heterotopias	D054221	Leukemia	1836
D008223	Epstein-barr virus infections	D020031	Lymphoma	2985
D005334	Fever	D008223	Lymphoma	1878
D005334	Fever	D007938	Leukemia	1184
D008223	Genetic diseases, inborn	D030342	Lymphoma	25,278
D007938	Genetic diseases, inborn	D030342	Leukemia	21,078
D009101	Genetic diseases, inborn	D030342	Multiple Myeloma	17,621
D006086	Graft versus host disease	D007938	Leukemia	1982
D006402	Hematologic diseases	D007938	Leukemia	1751
D008223	HIV infections	D015658	Lymphoma	1939
D007938	Myelodysplastic syndromes	D009190	Leukemia	2943
D009101	Renal insufficiency	D051437	Multiple Myeloma	1356
D007938	Thrombocytopenia	D013921	Leukemia	1012

DisGeNET				
Identifier	Related disease	Identifier	Blood cancer type	Jaccard index
C0004364	Autoimmune Diseases	C0024299	Lymphoma	0.24
C0023418	Myelodysplastic Syndrome	C3463824	Leukemia	0.25
C0003873	Rheumatoid Arthritis	C0023418	Leukemia	0.26
C0003873	Rheumatoid Arthritis	C0026764	Multiple Myeloma	0.25

## Discussion

Herein, we presented SicknessMiner, a TM pipeline encompassing our view towards a useful NLP approach for biomedical texts. Our approach highlights the usefulness of TM pipelines as integrated sources of information and as end-to-end platforms easy-accessible for beginner users. The use of individual modules in the pipeline allows us to easily replace and further optimize or introduce any enhancements independently, as new SOTA tools become available, which further improves the results of the pipeline as a whole. Furthermore, by using the BioBERT SOTA neural network module, we can take advantage of pre-trained weights that are made available, by the community, and that can be applied to different tasks beside NER (e.g. Relation Extraction and Question Answering) [17], some of which we would like to explore in future works. Moreover, SicknessMiner benefits from using BioBERT, since transfer learning is a powerful ML/DL technique that gains from any improved weights that arise in the community.

By using only abstracts rather than full-texts, our aim was to lower the false-positive rate for non-existent DDAs that could occur from negative results published in the full-text reports. Also, this enables a reproducible effect by resorting only to publicly available data since full-text articles are often difficult to access due to the existent publication fees. Despite some authors ponting to higher performance when using full texts instead of abstracts-only TM [26], the fact that the access to papers is not universal, justifies our choice to evaluate our results based on abstracts-only. Nonetheless, we also believe that to postulate new knowledge, one must always dedicate some human curatorship to the hypothesis at hand, which should resolve any errors brought by abstracts-only mining.

With an easy access GitHub repository (https://github.com/MoreiraLAB/SicknessMiner) and reproducible examples, SicknessMiner is a valuable tool for the NER and NEN steps of any TM pipeline for disease retrieval. Furthermore, SicknessMiner, via its co-mention analysis, is also able to successfully establish DDAs. Despite the fact that co-mention is often referred to as a lesser approach for establishing associations between mapped entities, SicknessMiner was able to recover close to 92% of DDAs from a well-established web server such as DisGeNET and still contribute with 16% of new DDAs. These pave the way for further analysis to assess new contributions to the field of BCs and related diseases. DisGeNET also includes DDAs from TM approaches and yet its performance is not improved compared to SicknessMiner. Moreover, DisGeNET needs gene or variant lists related to a disease, which can be tiresome and costly.

When analyzing our graphs (Figs. 2 and 3), the most noticeable aspect was that the majority of results obtained were cancers (for full results tables, see Supporting Information). Cancer is an intricate process that, despite our scientific advances, still lacks a complete thorough understanding. Indeed, cancer can be seen as a systemic process that possesses many layers in need of a system’s biology approach [27]. With this in mind, we focused instead on the most frequent co-mentioned or the ones with the highest Jaccard index non-cancerous diseases in the case of SicknessMiner and DisGeNET, respectively. SicknessMiner was able to retrieve nearly 6 times more non-cancerous diseases. In line with the systemic take on cancer, we also believe that the results retrieved solely by SicknessMiner as “anemia”, “fever”, and “thrombocytopenia” are encompassed in such a way that can grant a preview in the usefulness of such approaches. The only DDA reported by DisGeNET not present in the top 100 edges from SicknessMiner was rheumatoid arthritis that was assessed as associated with both leukemia and multiple myeloma. However, SicknessMiner also retrieved AutoImmune Diseases (AID) MeSH term, for which rheumatoid arthritis in one [28], in the top co-mentions. Finally, it is noteworthy to mention that the top 30% results retrieved from DisGeNET are some form or variation of the original query, as “childhood leukemia” or “adult lymphoma” whereas SicknessMiner, comparatively, retrieves less of those variations of the original query, highlighting “genetic diseases, inborn”, “epstein-barr virus infections”, and “myelodysplastic syndromes” in the top 30 edges. Nonetheless, the broad scope of non-cancerous DDAs found by SicknessMiner can provide a wider, more thorough glimpse and complement research lines by providing new insights of relevant DDAs.

During our work, the major problem regarding the NEN step, was the existence of several ontologies for diseases with the most eminent being DO [29], MeSH [30], and Unified Medical Language System Concept Unique Identifiers (UMLS CUI) [31]. In fact, results comparison can be hindered by the existence of several ontologies for which a direct correspondence or mapping is not always possible. A centralized ontology or the availability of an external mapping tool would be highly beneficial for TM pipelines both by enabling comparison, but also by allowing the integration of data from different sources onto the same model. Hence, in the future, other categories will be included in our model to fully take advantage of such impressive results in the DDA category, and to pave a new way towards accessibility in the AI-OMICS-era.

## Conclusion

Accurate TM solutions/software’s are sparse and still lack the inclusion of ML/DL models. Herein, we introduced SicknessMiner, a novel tool encompassing TM SOTA methods to simultaneously perform NER and NEN. SicknessMiner shows a very high performance and can retrieve 92% of all associations fetched by DisGeNET, a respected resource for DDAs. Despite the usefulness of obtaining relationships among diseases, most available models to tackle this problem tend to encompass a layer of ontology or some type of knowledge-based methodology. Our approach, despite seeming simpler, aims to harness the already available knowledge enclosed in scientific papers and uses text-mining tools to retrieve DDAs. We believe that through the development of SicknessMiner, we were able to build a comprehensive, highly upgradeable and customizable, easy to use TM pipeline to postulate new relevant DDAs.

## Methods

### TM approach: building sicknessminer

We used SOTA modules as the basis for the two tasks: BioBERT [17] for NER and NormCo [19] for NEN. BioBERT is a Deep Neural Network (DNN) model, a biomedical domain application of the Bidirectional Encoder Representations from Transformers (BERT) model [32]. BERT models have shown promising performances in a variety of NLP tasks, including NER. BioBERT provides different sets of weights^3^ to initialize the neural network, but the best results require parameter training on a distribution similar to the target distribution. We fitted the BioBERT parameters on the NCBI Disease dataset.^4^ A similar approach was performed for the NEN task using as basis the NormCo [19], a simple, yet powerful, model that uses Recurrent Neural Networks (RNN) to map the name of the entities into the IDs in a given ontology. We started from a pre-trained set of weights, made available by the authors of NormCo,^5^ and fine-tuning the model to the NCBI Disease Corpus [18].

Both NER and NEN fine-tuning were performed following the experiments described in the corresponding papers, and code provided by the authors. In particular, the NER system was implemented using TensorFlow,^6^ an Adam optimizer with a decay rate of 0.01, and a learning rate of 5e^−5^ trained for 10 epochs. The NEN system was implemented using Torch,^7^ with the same optimizer. We used a learning rate of 5e^−4^ and 100 epochs. After each epoch, the model was evaluated against a validation set (a set of examples distinct from the training and test sets) and stopped if no improvement of the model was achieved after 15 epochs.

### SicknessMiner evaluation

The evaluation of SicknessMiner was performed in the test set of the NCBI Dataset using the BC5CDR evaluation kit to compute the precision, recall and F1-score for both NER and NEN. This evaluation kit is commonly used to evaluate such tasks since it employs the widely used BC5CDR corpus for results comparison [19]. The NEN was performed on the result of the NER module, which means that errors from NER may cascade to NEN. We opted to use the BC5CDR evaluation kit, instead of the reported evaluation result implemented by each system, to attain a consistent evaluation across all systems. The precision is given by the formula $$P = \frac{tp}{tp + fp}$$, where $$tp$$ is the number of true positives and $$fp$$ is the number of false positives. The recall is given by the formula $$R = \frac{tp}{tp + fn}$$, where $$fn$$ is the number of false negatives. Finally, the F1-score is given by the formula $$F = \frac{2*P*R}{P+R}$$. Intuitively, the precision measures the confidence of the model in predicting the true examples, whilst the recall measures the capacity of the model to detect the true examples among all data; and the F1-score is a harmonic mean between the precision and recall, used to summarize both metrics in a single number, biased toward the smaller value between the two metrics.

### DDAs retrieval

PubMed used queries were: “((leukemia[Title/Abstract]) OR (multiple myeloma[Title/Abstract])) OR (lymphoma[Title/Abstract])”, giving a total of 390 K titles and abstracts (as of 21st of April 2021). SicknessMiner was compared to DisGeNET with the Concept IDs for the BCs types: C0023418, C0024299, and C0026764 for leukemia, lymphoma and multiple myeloma, respectively. Upon results collection, and since DisGeNET and SicknessMiner use different ontologies to identify diseases, an extra mapping step was performed to match results from both. DisGeNET uses the UMLS CUI and SicknessMiner uses both MeSH and OMIM identifiers. One caveat of mapping at the NEN step was related to the mismatch among the ontologies used by different sources that difficulties results’ comparison and/or combination. In order to overcome this shortcoming and to easily compare SicknessMiner and DisGeNET, we converted the DisGeNET mappings, originally in UMLS CUI, to MeSH or OMIM identifiers. To increase the identification power of our approach and to include additional identifiers descriptors, further mapping was performed recursively searching the xml files desc2021, supp2021, pa2021 and qual2021 made available via FTP by NCBI (https://nlmpubs.nlm.nih.gov/projects/mesh/MESH_FILES/xmlmesh/). All performed mappings are also available on our GitHub repository.

### DDAs evaluation

SicknessMiner was qualitatively and quantitatively compared against the results obtained through DisGeNET by both graph representation and similarity/dissimilarity percentage calculations. The set of genes of the target and related disease were sorted using the Jaccard index, a measurement of similarity between two different sets (A and B), given by the formula $\frac{|A\cap B|}{|A\cup B|}$.

Depending on the methodology, we considered that diseases were related in two different ways. For SicknessMiner, we considered that two diseases were positively related to each other whenever there was a co-mention amongst the paper title and/or abstract. Differently, for DisGeNET, we considered that two diseases were related whenever they shared, at least, 20 genes. Hence, relations were extracted for the entirety of the results, ranked according to the decrescent number of co-mentions or shared genes for SicknessMiner and DisGeNET, respectively, and the top 100 more frequent relations were kept for further analysis.

## Supplementary Information

**Additional file 1**. **Table 1** - SicknessMiner full results (organized by decreasing number of co-mentions).

## Data Availability

All data generated during this study are included in this paper and its respective Supplementary Information. Also, SicknessMiner is freely available under a GPLv3 license (https://www.gnu.org/licenses/gpl-3.0.en.html) on our lab GitHub (https://github.com/MoreiraLAB/SicknessMiner) and the models can be found on https://figshare.com/s/04259fac69da301680c2.

## Footnotes

1 SicknessMiner is publicly available at https://github.com/MoreiraLAB/SicknessMiner.

2 https://biocreative.bioinformatics.udel.edu/resources/corpora/biocreative-v-cdr-corpus/.

3 https://github.com/dmis-lab/biobert.

4 https://www.ncbi.nlm.nih.gov/CBBresearch/Dogan/DISEASE/.

5 https://github.com/IBM/aihn-ucsd/tree/master/NormCo-deep-disease-normalization.

6 https://www.tensorflow.org.

7 https://pytorch.org.

## References

[1] Batool Z, Usman M, Saleem K, Abdullah-Al-Wadud M, Fazal-e-Amin A-E (2018). Disease–disease association using network modeling: challenges and opportunities. J Med Imaging Health Inform.

[2] Opap K, Mulder N (2017). Recent advances in predicting gene-disease associations. F1000Res.

[3] Bello SM, Shimoyama M, Mitraka E, Laulederkind SJF, Smith CL, Eppig JT (2018). Disease ontology: improving and unifying disease annotations across species. Dis Model Mech.

[4] Piñero J, Ramírez-Anguita JM, Saüch-Pitarch J, Ronzano F, Centeno E, Sanz F (2020). The DisGeNET knowledge platform for disease genomics: 2019 update. Nucleic Acids Res.

[5] Zhu F, Patumcharoenpol P, Zhang C, Yang Y, Chan J, Meechai A (2013). Biomedical text mining and its applications in cancer research. J Biomed Inform.

[6] Lever J, Jones MR, Danos AM, Krysiak K, Bonakdar M, Grewal JK (2019). Text-mining clinically relevant cancer biomarkers for curation into the CIViC database. Genome Med.

[7] Pan Y, Zhang Y, Liu J (2018). Text mining-based drug discovery in cutaneous squamous cell carcinoma. Oncol Rep.

[8] Lin H-J, Sheu PC-Y, Tsai JJP, Wang CCN, Chou C-Y (2020). Text mining in a literature review of urothelial cancer using topic model. BMC Cancer.

[9] García Del Valle EP, Lagunes García G, Prieto Santamaría L, Zanin M, Menasalvas Ruiz E, Rodríguez-González A (2019). Disease networks and their contribution to disease understanding: a review of their evolution, techniques and data sources. J Biomed Inform.

[10] Miaocen Z (2020). A review on diagnosis and treatments of blood cancer. J Toxicol Environ Health B Crit Rev.

[11] Bray F, Ferlay J, Soerjomataram I, Siegel RL, Torre LA, Jemal A (2018). Global cancer statistics 2018: GLOBOCAN estimates of incidence and mortality worldwide for 36 cancers in 185 countries. CA Cancer J Clin.

[12] Hoehndorf R, Schofield PN, Gkoutos GV (2015). Analysis of the human diseasome using phenotype similarity between common, genetic, and infectious diseases. Sci Rep.

[13] van Driel MA, Bruggeman J, Vriend G, Brunner HG, Leunissen JAM (2006). A text-mining analysis of the human phenome. Eur J Hum Genet.

[14] Rosário-Ferreira N, Marques-Pereira C, Pires M, Ramalhão D, Pereira N, Guimarães V (2021). The treasury chest of text mining: piling available resources for powerful biomedical text mining. BioChem.

[15] Lamurias A, Couto FM. Text mining for bioinformatics using biomedical literature. In: Encyclopedia of bioinformatics and computational biology. Elsevier; 2019. p. 602–11.

[16] Zhao S, Su C, Lu Z, Wang F (2020). Recent advances in biomedical literature mining. Brief Bioinform.

[17] Lee J, Yoon W, Kim S, Kim D, Kim S, So CH (2020). BioBERT: a pre-trained biomedical language representation model for biomedical text mining. Bioinformatics.

[18] Doğan RI, Leaman R, Lu Z (2014). NCBI disease corpus: a resource for disease name recognition and concept normalization. J Biomed Inform.

[19] Wright D, Katsis Y, Mehta R, Hsu C-N. NormCo: deep disease normalization for biomedical knowledge base construction [Internet]. Automated Knowledge Base Construction (AKBC); 2019. https://www.akbc.ws/2019/papers/BJerQWcp6Q

[20] Suratanee A, Plaimas K (2015). DDA: a novel network-based scoring method to identify disease-disease associations. Bioinform Biol Insights.

[21] Le D-H, Pham V-H (2017). HGPEC: a Cytoscape app for prediction of novel disease-gene and disease-disease associations and evidence collection based on a random walk on heterogeneous network. BMC Syst Biol.

[22] Žitnik M, Janjić V, Larminie C, Zupan B, Pržulj N (2013). Discovering disease-disease associations by fusing systems-level molecular data. Sci Rep.

[23] Lee D-S, Park J, Kay KA, Christakis NA, Oltvai ZN, Barabási A-L (2008). The implications of human metabolic network topology for disease comorbidity. Proc Natl Acad Sci U S A.

[24] Goh K-I, Cusick ME, Valle D, Childs B, Vidal M, Barabási A-L (2007). The human disease network. Proc Natl Acad Sci USA.

[25] Sun K, Gonçalves JP, Larminie C, Przulj N (2014). Predicting disease associations via biological network analysis. BMC Bioinformatics.

[26] Westergaard D, Stærfeldt H-H, Tønsberg C, Jensen LJ, Brunak S (2018). A comprehensive and quantitative comparison of text-mining in 15 million full-text articles versus their corresponding abstracts. PLoS Comput Biol.

[27] Paul D. The systemic hallmarks of cancer. J Cancer Metastasis Treat [Internet]. 2020 Aug 28;2020. https://jcmtjournal.com/article/view/3625

[28] Barcellini W, Giannotta JA, Fattizzo B (2021). Autoimmune complications in hematologic neoplasms. Cancers.

[29] Schriml LM, Mitraka E, Munro J, Tauber B, Schor M, Nickle L (2019). Human disease ontology 2018 update: classification, content and workflow expansion. Nucleic Acids Res.

[30] Medical Subject Headings—Home Page. 2020 Jul 23 [cited 2021 Jun 13]; https://www.nlm.nih.gov/mesh/meshhome.html

[31] Bodenreider O (2004). The Unified Medical Language System (UMLS): integrating biomedical terminology. Nucleic Acids Res.

[32] Devlin J, Chang M-W, Lee K, Toutanova K. In: Proceedings of the 2019 conference of the north [Internet]. Stroudsburg: Association for Computational Linguistics; 2019. http://aclweb.org/anthology/N19-1423

