# Supplementary material for: SicknessMiner: a deep-learning-driven text-mining tool to abridge disease-disease associations
Source: BMC Bioinformatics. 2021 Oct 4;22:482. doi: 10.1186/s12859-021-04397-w (PMC8491382; doi:10.1186/s12859-021-04397-w)
Supplement: Supplementary file 1 — Additional file 1. Table 1 - SicknessMiner full results (organized by decreasing number of co-mentions). [file 12859_2021_4397_MOESM1_ESM.pdf]

Streamlining Disease-Disease Associations Using a Deep-Learning-driven Text-Mining Approach

Nícia Rosário-Ferreira [1,2,‡*], Victor Guimarães [3,4,‡], Vitor S. Costa [3,4], Irina S. Moreira [5,6*]

[1] CQC - Coimbra Chemistry Center, Chemistry Department, Faculty of Science and Technology, University of Coimbra, 3004-535 Coimbra, Portugal

[2] CNC - Center for Neuroscience and Cell Biology, University of Coimbra, Coimbra, Portugal

[3] Department of Sciences, University of Porto, Porto, Portugal

[4] INESC-TEC - Centre of Advanced Computing Systems, Porto, Portugal

[5] Department of Life Sciences, University of Coimbra, Calçada Martim de Freitas, 3000-456 Coimbra, Portugal

[6] CNC - Center for Neuroscience and Cell Biology, CIBB - Center for Innovative Biomedicine and Biotechnology, University of Coimbra, Coimbra, Portugal

[‡] Co-first authors

[*] Correspondence: nicia.ferreira@student.uc.pt (NR-F) and irina.moreira@cnc.uc.pt (ISM)

Table 1 - SicknessMiner full results (organized by decreasing number of co-mentions)

SicknessMiner				
Identifier 1	**Name 1**	***Identifier 2***	**Name 2**	**Co-mentions**
D008223	Lymphoma	*D009369*	Neoplasms	31915
D007938	Leukemia	*D009369*	Neoplasms	28614
D008223	Lymphoma	*D030342*	Genetic Diseases, Inborn	25278
D007938	Leukemia	*D030342*	Genetic Diseases, Inborn	21078
D009101	Multiple Myeloma	*D030342*	Genetic Diseases, Inborn	17621
D007938	Leukemia	*D015470*	Leukemia, Myeloid, Acute	17065
D008223	Lymphoma	*D008228*	Lymphoma, Non-Hodgkin	14826
D007938	Leukemia	*D054198*	Precursor Cell Lymphoblastic Leukemia-Lymphoma	12625
D009101	Multiple Myeloma	*D009369*	Neoplasms	12205
D007938	Leukemia	*D008223*	Lymphoma	8721
D008223	Lymphoma	*D016403*	Lymphoma, Large B-Cell, Diffuse	7768
D007938	Leukemia	*D015464*	Leukemia, Myelogenous, Chronic, BCR-ABL Positive	7289
D006689	Hodgkin Disease	*D008223*	Lymphoma	6205
D008223	Lymphoma	*D016393*	Lymphoma, B-Cell	5987
D007938	Leukemia	*D019337*	Hematologic Neoplasms	5124

D008223	Lymphoma	*D008224*	Lymphoma, Follicular	4816
D008223	Lymphoma	*D016399*	Lymphoma, T-Cell	4667
D008223	Lymphoma	*605027*	-	4618
D007938	Leukemia	*D015451*	Leukemia, Lymphocytic, Chronic, B-Cell	4336
D008223	Lymphoma	*D015451*	Leukemia, Lymphocytic, Chronic, B-Cell	4179
D008223	Lymphoma	*D020522*	Lymphoma, Mantle-Cell	3533
D007938	Leukemia	*D007951*	Leukemia, Myeloid	3528
D008223	Lymphoma	*D018442*	Lymphoma, B-Cell, Marginal Zone	3256
D008223	Lymphoma	*D008232*	Lymphoproliferative Disorders	2993
D008223	Lymphoma	*D020031*	Epstein-Barr Virus Infections	2985
D008223	Lymphoma	*D009101*	Multiple Myeloma	2983
D007938	Leukemia	*D009190*	Myelodysplastic Syndromes	2943
D008223	Lymphoma	*D019337*	Hematologic Neoplasms	2921
D002051	Burkitt Lymphoma	*D008223*	Lymphoma	2884
D002869	Chromosome Aberrations	*D007938*	Leukemia	2834
D001943	Breast Neoplasms	*D007938*	Leukemia	2734
D007938	Leukemia	*D015473*	Leukemia, Promyelocytic, Acute	2727
D009101	Multiple Myeloma	*D019337*	Hematologic Neoplasms	2715
D007938	Leukemia	*D008228*	Lymphoma, Non-Hodgkin	2687
D007938	Leukemia	*D054218*	Precursor T-Cell Lymphoblastic Leukemia-Lymphoma	2597
D008223	Lymphoma	*D015458*	Leukemia, T-Cell	2387
D001943	Breast Neoplasms	*D008223*	Lymphoma	2361
D000163	Acquired Immunodeficiency Syndrome	*D008223*	Lymphoma	2358
D007938	Leukemia	*D009101*	Multiple Myeloma	2329
D009101	Multiple Myeloma	*D010265*	Paraproteinemias	2212
D008223	Lymphoma	*D017728*	Lymphoma, Large-Cell, Anaplastic	2194
D008223	Lymphoma	*D054198*	Precursor Cell Lymphoblastic Leukemia-Lymphoma	2134
D008206	Lymphatic Diseases	*D008223*	Lymphoma	2090
D002277	Carcinoma	*D008223*	Lymphoma	2027
D006086	Graft vs Host Disease	*D007938*	Leukemia	1982
D008228	Lymphoma, Non-Hodgkin	*D009101*	Multiple Myeloma	1948
D008223	Lymphoma	*D015658*	HIV Infections	1939
D007938	Leukemia	*D007945*	Leukemia, Lymphoid	1906
D005334	Fever	*D008223*	Lymphoma	1878
D007938	Leukemia	*D054221*	Classical Lissencephalies and Subcortical Band Heterotopias	1836
D007938	Leukemia	*D015458*	Leukemia, T-Cell	1812
D007938	Leukemia	*D015456*	Leukemia, Biphenotypic, Acute	1784
D006402	Hematologic Diseases	*D007938*	Leukemia	1751
D001327	Autoimmune Diseases	*D008223*	Lymphoma	1680
D008223	Lymphoma	*D015470*	Leukemia, Myeloid, Acute	1633
D008175	Lung Neoplasms	*D008223*	Lymphoma	1599
D001847	Bone Diseases	*D009101*	Multiple Myeloma	1548

D008223	Lymphoma	*D008545*	Melanoma	1524
D008223	Lymphoma	*D015459*	Leukemia-Lymphoma, Adult T-Cell	1521
D008223	Lymphoma	*D016410*	Lymphoma, T-Cell, Cutaneous	1489
D007938	Leukemia	*D007948*	Leukemia, Monocytic, Acute	1478
D009101	Multiple Myeloma	*D010954*	Plasmacytoma	1469
D006689	Hodgkin Disease	*D007938*	Leukemia	1434
D007938	Leukemia	*D008545*	Melanoma	1381
D007938	Leukemia	*D008175*	Lung Neoplasms	1379
D008223	Lymphoma	*D016411*	Lymphoma, T-Cell, Peripheral	1373
D009101	Multiple Myeloma	*D051437*	Renal Insufficiency	1356
D004194	Disease	*D008223*	Lymphoma	1346
D003110	Colonic Neoplasms	*D008223*	Lymphoma	1318
D009101	Multiple Myeloma	*D015451*	Leukemia, Lymphocytic, Chronic, B-Cell	1297
D008223	Lymphoma	*D012509*	Sarcoma	1284
D007938	Leukemia	*D009503*	Neutropenia	1253
D008223	Lymphoma	*D054391*	Lymphoma, Extranodal NK-T-Cell	1219
D008998	Monoclonal Gammopathy of Undetermined Significance	*D009101*	Multiple Myeloma	1215
D001943	Breast Neoplasms	*D009101*	Multiple Myeloma	1197
D004194	Disease	*D007938*	Leukemia	1185
D005334	Fever	*D007938*	Leukemia	1184
D007938	Leukemia	*D012509*	Sarcoma	1164
D002869	Chromosome Aberrations	*D008223*	Lymphoma	1161
D000686	Amyloidosis	*D009101*	Multiple Myeloma	1108
D007938	Leukemia	*D010051*	Ovarian Neoplasms	1099
D008223	Lymphoma	*D013274*	Stomach Neoplasms	1077
D007938	Leukemia	*D009196*	Myeloproliferative Disorders	1071
D008223	Lymphoma	*D015464*	Leukemia, Myelogenous, Chronic, BCR-ABL Positive	1056
D007938	Leukemia	*D015448*	Leukemia, B-Cell	1056
D008223	Lymphoma	*D016543*	Central Nervous System Neoplasms	1054
D006689	Hodgkin Disease	*D009101*	Multiple Myeloma	1047
D007938	Leukemia	*D016399*	Lymphoma, T-Cell	1041
D003110	Colonic Neoplasms	*D007938*	Leukemia	1024
D009101	Multiple Myeloma	*D015470*	Leukemia, Myeloid, Acute	1023
D007945	Leukemia, Lymphoid	*D008223*	Lymphoma	1013
D007938	Leukemia	*D013921*	Thrombocytopenia	1012
D000230	Adenocarcinoma	*D008223*	Lymphoma	1007
D000740	Anemia	*D007938*	Leukemia	992
D008223	Lymphoma	*D012514*	Sarcoma, Kaposi	969
D008223	Lymphoma	*D016609*	Neoplasms, Second Primary	944
D007938	Leukemia	*D016393*	Lymphoma, B-Cell	910
D008223	Lymphoma	*D009503*	Neutropenia	909
C538324	3-Hydroxy-3-Methylglutaryl-CoA Lyase Deficiency	*D008223*	Lymphoma	907

D000163	Acquired Immunodeficiency Syndrome	*D007938*	Leukemia	906
D008223	Lymphoma	*D010051*	Ovarian Neoplasms	901
D008223	Lymphoma	*D013577*	Syndrome	898
D009101	Multiple Myeloma	*D054219*	Neoplasms, Plasma Cell	890
D002294	Carcinoma, Squamous Cell	*D008223*	Lymphoma	874
D000740	Anemia	*D009101*	Multiple Myeloma	867
D000741	Anemia, Aplastic	*D007938*	Leukemia	866
D007938	Leukemia	*D055728*	Primary Myelofibrosis	863
D008223	Lymphoma	*D013921*	Thrombocytopenia	838
D001932	Brain Neoplasms	*D007938*	Leukemia	832
D007674	Kidney Diseases	*D009101*	Multiple Myeloma	828
D001260	Ataxia Telangiectasia	*D008223*	Lymphoma	827
D001260	Ataxia Telangiectasia	*D007938*	Leukemia	820
D002051	Burkitt Lymphoma	*D007938*	Leukemia	819
D007938	Leukemia	*D016609*	Neoplasms, Second Primary	806
D002869	Chromosome Aberrations	*D009101*	Multiple Myeloma	806
D009101	Multiple Myeloma	*D015464*	Leukemia, Myelogenous, Chronic, BCR-ABL Positive	805
D002277	Carcinoma	*D007938*	Leukemia	795
D007239	Infections	*D007938*	Leukemia	766
D001932	Brain Neoplasms	*D008223*	Lymphoma	762
D001855	Bone Marrow Diseases	*D007938*	Leukemia	736
D007938	Leukemia	*D011471*	Prostatic Neoplasms	731
D009101	Multiple Myeloma	*D054198*	Precursor Cell Lymphoblastic Leukemia-Lymphoma	721
D009101	Multiple Myeloma	*D013921*	Thrombocytopenia	712
D007938	Leukemia	*D008232*	Lymphoproliferative Disorders	710
D007938	Leukemia	*D016403*	Lymphoma, Large B-Cell, Diffuse	703
D007154	Immune System Diseases	*D008223*	Lymphoma	703
D000208	Acute Disease	*D007938*	Leukemia	703
D008107	Liver Diseases	*D008223*	Lymphoma	701
D006528	Carcinoma, Hepatocellular	*D007938*	Leukemia	694
D002908	Chronic Disease	*D007938*	Leukemia	694
D006934	Hypercalcemia	*D009101*	Multiple Myeloma	693
D008171	Lung Diseases	*D008223*	Lymphoma	690
D007938	Leukemia	*D018805*	Sepsis	687
D008223	Lymphoma	*D015179*	Colorectal Neoplasms	682
D001172	Arthritis, Rheumatoid	*D008223*	Lymphoma	677
D006402	Hematologic Diseases	*D008223*	Lymphoma	676
D007938	Leukemia	*605027*	-	673
D007938	Leukemia	*D013163*	Splenomegaly	667
D008223	Lymphoma	*D015448*	Leukemia, B-Cell	664
D000740	Anemia	*D008223*	Lymphoma	663
D008223	Lymphoma	*D013163*	Splenomegaly	661
D004194	Disease	*D009101*	Multiple Myeloma	659

D008223	Lymphoma	*D054218*	Precursor T-Cell Lymphoblastic Leukemia-Lymphoma	654
D007938	Leukemia	*D009447*	Neuroblastoma	652
D008223	Lymphoma	*D012878*	Skin Neoplasms	648
D008258	Waldenstrom Macroglobulinemia	*D009101*	Multiple Myeloma	641
D007938	Leukemia	*D007943*	Leukemia, Hairy Cell	633
D008232	Lymphoproliferative Disorders	*D009101*	Multiple Myeloma	625
D007938	Leukemia	*D015459*	Leukemia-Lymphoma, Adult T-Cell	625
D009101	Multiple Myeloma	*D009503*	Neutropenia	623
D008223	Lymphoma	*D012595*	Scleroderma, Systemic	600
D006528	Carcinoma, Hepatocellular	*D008223*	Lymphoma	596
D009101	Multiple Myeloma	*605027*	-	595
D006333	Heart Failure	*D008223*	Lymphoma	588
D007938	Leukemia	*D015658*	HIV Infections	582
D008223	Lymphoma	*D009447*	Neuroblastoma	577
D008223	Lymphoma	*D008258*	Waldenstrom Macroglobulinemia	577
D006402	Hematologic Diseases	*D009101*	Multiple Myeloma	572
D008223	Lymphoma	*D011014*	Pneumonia	566
D007951	Leukemia, Myeloid	*D008223*	Lymphoma	559
D007938	Leukemia	*D015179*	Colorectal Neoplasms	554
D009101	Multiple Myeloma	*D009190*	Myelodysplastic Syndromes	553
D007938	Leukemia	*D011014*	Pneumonia	546
D008223	Lymphoma	*D009190*	Myelodysplastic Syndromes	543
D007674	Kidney Diseases	*D008223*	Lymphoma	536
D007938	Leukemia	*D013274*	Stomach Neoplasms	530
D006086	Graft vs Host Disease	*D008223*	Lymphoma	515
D008223	Lymphoma	*D011471*	Prostatic Neoplasms	514
D008223	Lymphoma	*D010265*	Paraproteinemias	509
D007239	Infections	*D008223*	Lymphoma	504
D001327	Autoimmune Diseases	*D007938*	Leukemia	498
D009101	Multiple Myeloma	*D011471*	Prostatic Neoplasms	497
D008175	Lung Neoplasms	*D009101*	Multiple Myeloma	493
D005221	Fatigue	*D009101*	Multiple Myeloma	487
D009101	Multiple Myeloma	*D016609*	Neoplasms, Second Primary	474
D007938	Leukemia	*D016543*	Central Nervous System Neoplasms	473
D008223	Lymphoma	*D018805*	Sepsis	466
D006333	Heart Failure	*D007938*	Leukemia	463
D007943	Leukemia, Hairy Cell	*D008223*	Lymphoma	462
D007938	Leukemia	*D008206*	Lymphatic Diseases	454
D009101	Multiple Myeloma	*D016393*	Lymphoma, B-Cell	453
D007938	Leukemia	*D020031*	Epstein-Barr Virus Infections	447
D001172	Arthritis, Rheumatoid	*D007938*	Leukemia	445
D008223	Lymphoma	*D010146*	Pain	440
D007938	Leukemia	*D008107*	Liver Diseases	438

D005221	Fatigue	*D008223*	Lymphoma	433
D008223	Lymphoma	*D010954*	Plasmacytoma	430
D003110	Colonic Neoplasms	*D009101*	Multiple Myeloma	430
D007154	Immune System Diseases	*D007938*	Leukemia	429
D009101	Multiple Myeloma	*D016403*	Lymphoma, Large B-Cell, Diffuse	419
D008545	Melanoma	*D009101*	Multiple Myeloma	419
D007938	Leukemia	*D013577*	Syndrome	417
D009101	Multiple Myeloma	*D012595*	Scleroderma, Systemic	404
D006086	Graft vs Host Disease	*D009101*	Multiple Myeloma	402
D007938	Leukemia	*D008224*	Lymphoma, Follicular	393
D009101	Multiple Myeloma	*D010146*	Pain	391
D008223	Lymphoma	*D051437*	Renal Insufficiency	390
D009101	Multiple Myeloma	*D020522*	Lymphoma, Mantle-Cell	384
D007938	Leukemia	*D020522*	Lymphoma, Mantle-Cell	381
D007938	Leukemia	*D008171*	Lung Diseases	361
D002908	Chronic Disease	*D008223*	Lymphoma	361
D005334	Fever	*D009101*	Multiple Myeloma	353
D007938	Leukemia	*D012878*	Skin Neoplasms	348
D006333	Heart Failure	*D009101*	Multiple Myeloma	325
D002294	Carcinoma, Squamous Cell	*D007938*	Leukemia	319
D009101	Multiple Myeloma	*D010051*	Ovarian Neoplasms	308
D001327	Autoimmune Diseases	*D009101*	Multiple Myeloma	302
D000230	Adenocarcinoma	*D007938*	Leukemia	297
D001855	Bone Marrow Diseases	*D008223*	Lymphoma	296
D006934	Hypercalcemia	*D008223*	Lymphoma	295
D001172	Arthritis, Rheumatoid	*D009101*	Multiple Myeloma	293
D008224	Lymphoma, Follicular	*D009101*	Multiple Myeloma	284
D007938	Leukemia	*D016410*	Lymphoma, T-Cell, Cutaneous	281
D005221	Fatigue	*D007938*	Leukemia	276
D000208	Acute Disease	*D008223*	Lymphoma	272
D007938	Leukemia	*D010146*	Pain	269
D002277	Carcinoma	*D009101*	Multiple Myeloma	269
D007938	Leukemia	*D051437*	Renal Insufficiency	262
D002051	Burkitt Lymphoma	*D009101*	Multiple Myeloma	259
D007674	Kidney Diseases	*D007938*	Leukemia	246
D009101	Multiple Myeloma	*D055728*	Primary Myelofibrosis	242
D007154	Immune System Diseases	*D009101*	Multiple Myeloma	240
D001855	Bone Marrow Diseases	*D009101*	Multiple Myeloma	240
D008223	Lymphoma	*D055728*	Primary Myelofibrosis	238
D008223	Lymphoma	*D054221*	Classical Lissencephalies and Subcortical Band Heterotopias	238
D000163	Acquired Immunodeficiency Syndrome	*D009101*	Multiple Myeloma	234
D000686	Amyloidosis	*D008223*	Lymphoma	233
D009101	Multiple Myeloma	*D011014*	Pneumonia	232

D008223	Lymphoma	*D009196*	Myeloproliferative Disorders	231
D007938	Leukemia	*D012595*	Scleroderma, Systemic	231
D001847	Bone Diseases	*D008223*	Lymphoma	227
D009101	Multiple Myeloma	*D013577*	Syndrome	224
D009101	Multiple Myeloma	*D015179*	Colorectal Neoplasms	223
D008223	Lymphoma	*D015456*	Leukemia, Biphenotypic, Acute	220
D007951	Leukemia, Myeloid	*D009101*	Multiple Myeloma	216
D009101	Multiple Myeloma	*D054221*	Classical Lissencephalies and Subcortical Band Heterotopias	212
D009101	Multiple Myeloma	*D015658*	HIV Infections	208
D002908	Chronic Disease	*D009101*	Multiple Myeloma	202
D006934	Hypercalcemia	*D007938*	Leukemia	198
D007938	Leukemia	*D017728*	Lymphoma, Large-Cell, Anaplastic	196
D009101	Multiple Myeloma	*D016399*	Lymphoma, T-Cell	188
D008107	Liver Diseases	*D009101*	Multiple Myeloma	187
D000741	Anemia, Aplastic	*D008223*	Lymphoma	182
D007938	Leukemia	*D010265*	Paraproteinemias	181
D007945	Leukemia, Lymphoid	*D009101*	Multiple Myeloma	179
D008223	Lymphoma	*D015473*	Leukemia, Promyelocytic, Acute	177
D009101	Multiple Myeloma	*D013274*	Stomach Neoplasms	175
D007938	Leukemia	*D016411*	Lymphoma, T-Cell, Peripheral	175
D007239	Infections	*D009101*	Multiple Myeloma	175
D009101	Multiple Myeloma	*D018805*	Sepsis	169
D009101	Multiple Myeloma	*D009196*	Myeloproliferative Disorders	169
D009101	Multiple Myeloma	*D020031*	Epstein-Barr Virus Infections	163
D007943	Leukemia, Hairy Cell	*D009101*	Multiple Myeloma	162
D009101	Multiple Myeloma	*D012509*	Sarcoma	160
D006528	Carcinoma, Hepatocellular	*D009101*	Multiple Myeloma	160
D000208	Acute Disease	*D009101*	Multiple Myeloma	156
D009101	Multiple Myeloma	*D012514*	Sarcoma, Kaposi	153
D007938	Leukemia	*D010954*	Plasmacytoma	147
D007948	Leukemia, Monocytic, Acute	*D008223*	Lymphoma	145
D007938	Leukemia	*D018442*	Lymphoma, B-Cell, Marginal Zone	144
D001847	Bone Diseases	*D007938*	Leukemia	144
D008223	Lymphoma	*D054219*	Neoplasms, Plasma Cell	140
D009101	Multiple Myeloma	*D018442*	Lymphoma, B-Cell, Marginal Zone	137
D008171	Lung Diseases	*D009101*	Multiple Myeloma	135
D002294	Carcinoma, Squamous Cell	*D009101*	Multiple Myeloma	132
D001932	Brain Neoplasms	*D009101*	Multiple Myeloma	120
D008223	Lymphoma	*D008998*	Monoclonal Gammopathy of Undetermined Significance	119
D009101	Multiple Myeloma	*D012878*	Skin Neoplasms	112
D000741	Anemia, Aplastic	*D009101*	Multiple Myeloma	110
D007938	Leukemia	*D012514*	Sarcoma, Kaposi	108
D009101	Multiple Myeloma	*D015473*	Leukemia, Promyelocytic, Acute	106

C538324	3-Hydroxy-3-Methylglutaryl-CoA Lyase Deficiency	*D009101*	Multiple Myeloma	105
C538324	3-Hydroxy-3-Methylglutaryl-CoA Lyase Deficiency	*D007938*	Leukemia	102
D009101	Multiple Myeloma	*D009447*	Neuroblastoma	100
D000230	Adenocarcinoma	*D009101*	Multiple Myeloma	96
D007938	Leukemia	*D008258*	Waldenstrom Macroglobulinemia	95
D009101	Multiple Myeloma	*D015458*	Leukemia, T-Cell	94
D007938	Leukemia	*D054391*	Lymphoma, Extranodal NK-T-Cell	94
D008206	Lymphatic Diseases	*D009101*	Multiple Myeloma	91
D009101	Multiple Myeloma	*D015448*	Leukemia, B-Cell	80
D009101	Multiple Myeloma	*D013163*	Splenomegaly	76
D001260	Ataxia Telangiectasia	*D009101*	Multiple Myeloma	71
D009101	Multiple Myeloma	*D054218*	Precursor T-Cell Lymphoblastic Leukemia-Lymphoma	70
D009101	Multiple Myeloma	*D017728*	Lymphoma, Large-Cell, Anaplastic	68
D009101	Multiple Myeloma	*D016410*	Lymphoma, T-Cell, Cutaneous	67
D009101	Multiple Myeloma	*D015459*	Leukemia-Lymphoma, Adult T-Cell	60
D007938	Leukemia	*D054219*	Neoplasms, Plasma Cell	57
D007948	Leukemia, Monocytic, Acute	*D009101*	Multiple Myeloma	46
D000686	Amyloidosis	*D007938*	Leukemia	45
D009101	Multiple Myeloma	*D016411*	Lymphoma, T-Cell, Peripheral	42
D007938	Leukemia	*D008998*	Monoclonal Gammopathy of Undetermined Significance	42
D009101	Multiple Myeloma	*D016543*	Central Nervous System Neoplasms	39
D009101	Multiple Myeloma	*D054391*	Lymphoma, Extranodal NK-T-Cell	29
D009101	Multiple Myeloma	*D015456*	Leukemia, Biphenotypic, Acute	22

Table 2 - DisGeNET full results (organized by decreasing number of Jaccard Index)

DisGeNET				
Identifier 1	**Name 1**	***Identifier 2***	**Name 2**	**Jaccard Index**
C0023418	Leukemia	*C1332977*	Childhood Leukemia	0.80
C0024299	Lymphoma	*C1332206*	Adult Lymphoma	0.76
C0024299	Lymphoma	*C1332979*	Childhood Lymphoma	0.75
C0023418	Leukemia	*C0023449*	Acute lymphocytic Leukemia	0.36
C0023418	Leukemia	*C0023467*	Leukemia. Myelocytic. Acute	0.35
C0023418	Leukemia	*C0024299*	Lymphoma	0.33
C0024299	Lymphoma	*C0079731*	B-Cell Lymphomas	0.33
C0024299	Lymphoma	*C1332977*	Childhood Leukemia	0.31
C0024299	Lymphoma	*C0079744*	Diffuse Large B-Cell Lymphoma	0.31
C0023434	Chronic Lymphocytic Leukemia	*C0026764*	Multiple Myeloma	0.31
C0024299	Lymphoma	*C0026764*	Multiple Myeloma	0.31
C0023418	Leukemia	*C0023452*	Childhood Acute Lymphoblastic Leukemia	0.31
C0023434	Chronic Lymphocytic Leukemia	*C0024299*	Lymphoma	0.30
C0023449	Acute lymphocytic Leukemia	*C0024299*	Lymphoma	0.30

C0023418	Leukemia	*C0023473*	Myeloid Leukemia. Chronic	0.30
C0024299	Lymphoma	*C0024305*	Lymphoma. Non-Hodgkin	0.29
C0023418	Leukemia	*C0598766*	Leukemogenesis	0.29
C0023418	Leukemia	*C0026764*	Multiple Myeloma	0.28
C0026764	Multiple Myeloma	*C0079731*	B-Cell Lymphomas	0.28
C0026764	Multiple Myeloma	*C1332206*	Adult Lymphoma	0.28
C0026764	Multiple Myeloma	*C1332979*	Childhood Lymphoma	0.28
C0023418	Leukemia	*C1332206*	Adult Lymphoma	0.28
C0023418	Leukemia	*C1332979*	Childhood Lymphoma	0.28
C0023418	Leukemia	*C0751606*	Adult Acute Lymphocytic Leukemia	0.28
C0023418	Leukemia	*C0025202*	melanoma	0.28
C0026764	Multiple Myeloma	*C1332977*	Childhood Leukemia	0.28
C0023418	Leukemia	*C0235974*	Pancreatic carcinoma	0.28
C0023418	Leukemia	*C0023434*	Chronic Lymphocytic Leukemia	0.27
C0023418	Leukemia	*C1961102*	Precursor Cell Lymphoblastic Leukemia Lymphoma	0.27
C0023418	Leukemia	*C0346647*	Malignant neoplasm of pancreas	0.27
C0023418	Leukemia	*C1621958*	Glioblastoma Multiforme	0.27
C0017636	Glioblastoma	*C0023418*	Leukemia	0.27
C0023452	Childhood Acute Lymphoblastic Leukemia	*C0024299*	Lymphoma	0.27
C0025202	melanoma	*C0026764*	Multiple Myeloma	0.27
C0023418	Leukemia	*C0079731*	B-Cell Lymphomas	0.27
C0007097	Carcinoma	*C0023418*	Leukemia	0.26
C0026764	Multiple Myeloma	*C0079744*	Diffuse Large B-Cell Lymphoma	0.26
C0007137	Squamous cell carcinoma	*C0023418*	Leukemia	0.26
C0023418	Leukemia	*C0027819*	Neuroblastoma	0.26
C0023418	Leukemia	*C1140680*	Malignant neoplasm of ovary	0.26
C0024299	Lymphoma	*C0598766*	Leukemogenesis	0.26
C0023418	Leukemia	*C0280474*	Childhood Glioblastoma	0.26
C0023418	Leukemia	*C0278878*	Adult Glioblastoma	0.26
C0023418	Leukemia	*C4721610*	Carcinoma. Ovarian Epithelial	0.26
C0023418	Leukemia	*C0699790*	Colon Carcinoma	0.26
C0023418	Leukemia	*C0700095*	Central neuroblastoma	0.26
C0023418	Leukemia	*C4086165*	Childhood Neuroblastoma	0.26
C0026764	Multiple Myeloma	*C0029463*	Osteosarcoma	0.26
C0003873	Rheumatoid Arthritis	*C0023418*	Leukemia	0.26
C0023418	Leukemia	*C0178874*	Tumor Progression	0.26
C0026764	Multiple Myeloma	*C1621958*	Glioblastoma Multiforme	0.26
C0023418	Leukemia	*C1458155*	Mammary Neoplasms	0.26
C0023467	Leukemia. Myelocytic. Acute	*C0026764*	Multiple Myeloma	0.25
C0023418	Leukemia	*C3463824*	MYELODYSPLASTIC SYNDROME	0.25
C0026764	Multiple Myeloma	*C0278878*	Adult Glioblastoma	0.25
C0026764	Multiple Myeloma	*C0585442*	Osteosarcoma of bone	0.25
C0026764	Multiple Myeloma	*C0280474*	Childhood Glioblastoma	0.25

C0023418	Leukemia	*C0919267*	ovarian neoplasm	0.25
C0026764	Multiple Myeloma	*C0235974*	Pancreatic carcinoma	0.25
C0019829	Hodgkin Disease	*C0024299*	Lymphoma	0.25
C0007134	Renal Cell Carcinoma	*C0023418*	Leukemia	0.25
C0026764	Multiple Myeloma	*C1332986*	Childhood Osteosarcoma	0.25
C0023449	Acute lymphocytic Leukemia	*C0026764*	Multiple Myeloma	0.25
C0026764	Multiple Myeloma	*C1168401*	Squamous cell carcinoma of the head and neck	0.25
C0026764	Multiple Myeloma	*C0279702*	Conventional (Clear Cell) Renal Cell Carcinoma	0.25
C0017636	Glioblastoma	*C0026764*	Multiple Myeloma	0.25
C0026764	Multiple Myeloma	*C0346647*	Malignant neoplasm of pancreas	0.25
C0024299	Lymphoma	*C0376545*	Hematologic Neoplasms	0.25
C0023418	Leukemia	*C1306460*	Primary malignant neoplasm of lung	0.25
C0001418	Adenocarcinoma	*C0023418*	Leukemia	0.25
C0023418	Leukemia	*C0279702*	Conventional (Clear Cell) Renal Cell Carcinoma	0.25
C0023473	Myeloid Leukemia. Chronic	*C0026764*	Multiple Myeloma	0.25
C0026764	Multiple Myeloma	*C2939419*	Secondary Neoplasm	0.25
C0007134	Renal Cell Carcinoma	*C0026764*	Multiple Myeloma	0.25
C0023418	Leukemia	*C0684249*	Carcinoma of lung	0.25
C0026764	Multiple Myeloma	*C0699885*	Carcinoma of bladder	0.25
C0007102	Malignant tumor of colon	*C0023418*	Leukemia	0.25
C0003873	Rheumatoid Arthritis	*C0026764*	Multiple Myeloma	0.25
C0007097	Carcinoma	*C0026764*	Multiple Myeloma	0.25
C0023418	Leukemia	*C0242379*	Malignant neoplasm of lung	0.25
C0024299	Lymphoma	*C0751606*	Adult Acute Lymphocytic Leukemia	0.25
C0023418	Leukemia	*C0600139*	Prostate carcinoma	0.25
C0005695	Bladder Neoplasm	*C0023418*	Leukemia	0.25
C0007131	Non-Small Cell Lung Carcinoma	*C0023418*	Leukemia	0.25
C0017638	Glioma	*C0023418*	Leukemia	0.25
C0023418	Leukemia	*C0376545*	Hematologic Neoplasms	0.25
C0023418	Leukemia	*C0029463*	Osteosarcoma	0.25
C0005695	Bladder Neoplasm	*C0026764*	Multiple Myeloma	0.24
C0023418	Leukemia	*C4722085*	Malignant neoplasm of colon and/or rectum	0.24
C0026764	Multiple Myeloma	*C0699790*	Colon Carcinoma	0.24
C0026764	Multiple Myeloma	*C0280100*	Solid Neoplasm	0.24
C0004364	Autoimmune Diseases	*C0024299*	Lymphoma	0.24
C0023418	Leukemia	*C0280100*	Solid Neoplasm	0.24
C0026764	Multiple Myeloma	*C4721610*	Carcinoma. Ovarian Epithelial	0.24
C0026764	Multiple Myeloma	*C0700095*	Central neuroblastoma	0.24
C0026764	Multiple Myeloma	*C4086165*	Childhood Neuroblastoma	0.24
C0023418	Leukemia	*C0376358*	Malignant neoplasm of prostate	0.24
C0005684	Malignant neoplasm of urinary bladder	*C0023418*	Leukemia	0.24
C0026764	Multiple Myeloma	*C0027819*	Neuroblastoma	0.24

C0023418	Leukemia	*C2939419*	Secondary Neoplasm	0.24
C0026764	Multiple Myeloma	*C1140680*	Malignant neoplasm of ovary	0.24
C0026764	Multiple Myeloma	*C0376545*	Hematologic Neoplasms	0.24
C0023467	Leukemia. Myelocytic. Acute	*C0024299*	Lymphoma	0.24
C0023418	Leukemia	*C0699885*	Carcinoma of bladder	0.24
C0023418	Leukemia	*C0699791*	Stomach Carcinoma	0.24
C0007137	Squamous cell carcinoma	*C0026764*	Multiple Myeloma	0.24
C0024299	Lymphoma	*C0280100*	Solid Neoplasm	0.24
C0005684	Malignant neoplasm of urinary bladder	*C0026764*	Multiple Myeloma	0.24
C0024141	Lupus Erythematosus. Systemic	*C0024299*	Lymphoma	0.24
C0026764	Multiple Myeloma	*C1458155*	Mammary Neoplasms	0.24
C0023473	Myeloid Leukemia. Chronic	*C0024299*	Lymphoma	0.24
C0001418	Adenocarcinoma	*C0026764*	Multiple Myeloma	0.24
C0007097	Carcinoma	*C0024299*	Lymphoma	0.24
C0007137	Squamous cell carcinoma	*C0024299*	Lymphoma	0.24
C0007131	Non-Small Cell Lung Carcinoma	*C0026764*	Multiple Myeloma	0.24
C0007102	Malignant tumor of colon	*C0026764*	Multiple Myeloma	0.24
C0023418	Leukemia	*C0585442*	Osteosarcoma of bone	0.24
C0023418	Leukemia	*C1332986*	Childhood Osteosarcoma	0.24
C0024299	Lymphoma	*C0025202*	melanoma	0.24
C0026764	Multiple Myeloma	*C1306460*	Primary malignant neoplasm of lung	0.24
C0004364	Autoimmune Diseases	*C0023418*	Leukemia	0.24
C0023418	Leukemia	*C0024623*	Malignant neoplasm of stomach	0.24
C0026764	Multiple Myeloma	*C0919267*	ovarian neoplasm	0.24
C0026764	Multiple Myeloma	*C4722085*	Malignant neoplasm of colon and/or rectum	0.24
C0026764	Multiple Myeloma	*C0178874*	Tumor Progression	0.24
C0026764	Multiple Myeloma	*C0242379*	Malignant neoplasm of lung	0.24
C0017638	Glioma	*C0026764*	Multiple Myeloma	0.24
C0003873	Rheumatoid Arthritis	*C0024299*	Lymphoma	0.24
C0026764	Multiple Myeloma	*C0684249*	Carcinoma of lung	0.24
C0026764	Multiple Myeloma	*C2931822*	Nasopharyngeal carcinoma	0.24
C0024299	Lymphoma	*C1961102*	Precursor Cell Lymphoblastic Leukemia Lymphoma	0.24
C0023418	Leukemia	*C0476089*	Endometrial Carcinoma	0.23
C0024299	Lymphoma	*C0235974*	Pancreatic carcinoma	0.23
C0023418	Leukemia	*C0596263*	Carcinogenesis	0.23
C0023418	Leukemia	*C0024141*	Lupus Erythematosus. Systemic	0.23
C0019196	Hepatitis C	*C0026764*	Multiple Myeloma	0.23
C0026764	Multiple Myeloma	*C3539878*	Triple Negative Breast Neoplasms	0.23
C0024299	Lymphoma	*C3463824*	MYELODYSPLASTIC SYNDROME	0.23
C0024299	Lymphoma	*C0346647*	Malignant neoplasm of pancreas	0.23
C0024299	Lymphoma	*C0027819*	Neuroblastoma	0.23
C0024299	Lymphoma	*C0700095*	Central neuroblastoma	0.23
C0024299	Lymphoma	*C4086165*	Childhood Neuroblastoma	0.23

C0007134	Renal Cell Carcinoma	*C0024299*	Lymphoma	0.23
C0026764	Multiple Myeloma	*C4722518*	Triple-Negative Breast Carcinoma	0.23
C0026764	Multiple Myeloma	*C4048328*	cervical cancer	0.23
C0023452	Childhood Acute Lymphoblastic Leukemia	*C0026764*	Multiple Myeloma	0.23
C0019196	Hepatitis C	*C0024299*	Lymphoma	0.23
C0026764	Multiple Myeloma	*C3463824*	MYELODYSPLASTIC SYNDROME	0.23
C0026764	Multiple Myeloma	*C0152013*	Adenocarcinoma of lung (disorder)	0.23
C0019163	Hepatitis B	*C0026764*	Multiple Myeloma	0.23
C0009402	Colorectal Carcinoma	*C0023418*	Leukemia	0.23
C0026764	Multiple Myeloma	*C0029408*	Degenerative polyarthritis	0.23
C0001418	Adenocarcinoma	*C0024299*	Lymphoma	0.23
C0023418	Leukemia	*C0042769*	Virus Diseases	0.23
C0026764	Multiple Myeloma	*C0600139*	Prostate carcinoma	0.23
C0023418	Leukemia	*C2239176*	Liver carcinoma	0.23
C0019829	Hodgkin Disease	*C0026764*	Multiple Myeloma	0.23
C0026764	Multiple Myeloma	*C0699791*	Stomach Carcinoma	0.23
C0023418	Leukemia	*C4048328*	cervical cancer	0.23
C0023418	Leukemia	*C0678222*	Breast Carcinoma	0.23
C0023418	Leukemia	*C1168401*	Squamous cell carcinoma of the head and neck	0.23
C0023418	Leukemia	*C0302592*	Cervix carcinoma	0.23
C0024299	Lymphoma	*C0042769*	Virus Diseases	0.23
C0026764	Multiple Myeloma	*C0042769*	Virus Diseases	0.23
C0019163	Hepatitis B	*C0024299*	Lymphoma	0.22
C0026764	Multiple Myeloma	*C0376358*	Malignant neoplasm of prostate	0.22
C0024299	Lymphoma	*C0279702*	Conventional (Clear Cell) Renal Cell Carcinoma	0.22
C0011849	Diabetes Mellitus	*C0023418*	Leukemia	0.22
C0004153	Atherosclerosis	*C0023418*	Leukemia	0.22
C0026764	Multiple Myeloma	*C0302592*	Cervix carcinoma	0.22
C0023418	Leukemia	*C0027627*	Neoplasm Metastasis	0.22
C0003850	Arteriosclerosis	*C0023418*	Leukemia	0.22
C0026764	Multiple Myeloma	*C0476089*	Endometrial Carcinoma	0.22
C0011847	Diabetes	*C0023418*	Leukemia	0.22
C0023418	Leukemia	*C0079744*	Diffuse Large B-Cell Lymphoma	0.22
C0004364	Autoimmune Diseases	*C0026764*	Multiple Myeloma	0.22
C0004153	Atherosclerosis	*C0026764*	Multiple Myeloma	0.22
C0003850	Arteriosclerosis	*C0026764*	Multiple Myeloma	0.22
C0026764	Multiple Myeloma	*C0345904*	Malignant neoplasm of liver	0.22
C0024623	Malignant neoplasm of stomach	*C0026764*	Multiple Myeloma	0.22
C0007847	Malignant tumor of cervix	*C0026764*	Multiple Myeloma	0.22
C0023418	Leukemia	*C0686619*	Secondary malignant neoplasm of lymph node	0.22
C0026764	Multiple Myeloma	*C1832661*	ANOPHTHALMIA AND PULMONARY HYPOPLASIA	0.22
C0023418	Leukemia	*C0033578*	Prostatic Neoplasms	0.22

C0006142	Malignant neoplasm of breast	*C0023418*	Leukemia	0.22
C0026764	Multiple Myeloma	*C0026769*	Multiple Sclerosis	0.22
C0017636	Glioblastoma	*C0024299*	Lymphoma	0.22
C0024299	Lymphoma	*C1140680*	Malignant neoplasm of ovary	0.22
C0024141	Lupus Erythematosus. Systemic	*C0026764*	Multiple Myeloma	0.22
C0024299	Lymphoma	*C1621958*	Glioblastoma Multiforme	0.22
C0023418	Leukemia	*C2931822*	Nasopharyngeal carcinoma	0.22
C0024299	Lymphoma	*C4721532*	Lymphoma. Non-Hodgkin. Familial	0.22
C0019196	Hepatitis C	*C0023418*	Leukemia	0.22
C0007847	Malignant tumor of cervix	*C0023418*	Leukemia	0.22
C0023418	Leukemia	*C0152013*	Adenocarcinoma of lung (disorder)	0.22
C0005695	Bladder Neoplasm	*C0024299*	Lymphoma	0.22
C0024299	Lymphoma	*C0029463*	Osteosarcoma	0.22
C0026764	Multiple Myeloma	*C0279626*	Squamous cell carcinoma of esophagus	0.22
C0023418	Leukemia	*C3539878*	Triple Negative Breast Neoplasms	0.22
C0024299	Lymphoma	*C2939419*	Secondary Neoplasm	0.22
C0023418	Leukemia	*C0279626*	Squamous cell carcinoma of esophagus	0.22
C0024299	Lymphoma	*C0699790*	Colon Carcinoma	0.22
C0024299	Lymphoma	*C1458155*	Mammary Neoplasms	0.22
C0024299	Lymphoma	*C0079772*	T-Cell Lymphoma	0.22
C0024299	Lymphoma	*C0280474*	Childhood Glioblastoma	0.22
C0026764	Multiple Myeloma	*C0686619*	Secondary malignant neoplasm of lymph node	0.22
C0024299	Lymphoma	*C0278878*	Adult Glioblastoma	0.22
C0024299	Lymphoma	*C0919267*	ovarian neoplasm	0.22
C0024299	Lymphoma	*C4721610*	Carcinoma. Ovarian Epithelial	0.22
C0024299	Lymphoma	*C0699885*	Carcinoma of bladder	0.22
C0023418	Leukemia	*C0024121*	Lung Neoplasms	0.22
C0024299	Lymphoma	*C0585442*	Osteosarcoma of bone	0.22
C0019163	Hepatitis B	*C0023418*	Leukemia	0.22
C0005684	Malignant neoplasm of urinary bladder	*C0024299*	Lymphoma	0.22
C0021051	Immunologic Deficiency Syndromes	*C0024299*	Lymphoma	0.21
C0023418	Leukemia	*C4722518*	Triple-Negative Breast Carcinoma	0.21
C0023418	Leukemia	*C1306459*	Primary malignant neoplasm	0.21
C0024299	Lymphoma	*C1332986*	Childhood Osteosarcoma	0.21
C0002395	Alzheimer's Disease	*C0023418*	Leukemia	0.21
C0024299	Lymphoma	*C2931822*	Nasopharyngeal carcinoma	0.21
C0009324	Ulcerative Colitis	*C0026764*	Multiple Myeloma	0.21
C0023418	Leukemia	*C1269955*	Tumor Cell Invasion	0.21
C0024299	Lymphoma	*C0476089*	Endometrial Carcinoma	0.21
C0024121	Lung Neoplasms	*C0024299*	Lymphoma	0.21
C0011849	Diabetes Mellitus	*C0026764*	Multiple Myeloma	0.21
C0026764	Multiple Myeloma	*C2239176*	Liver carcinoma	0.21
C0024299	Lymphoma	*C1168401*	Squamous cell carcinoma of the head and neck	0.21

C0024305	Lymphoma. Non-Hodgkin	*C0026764*	Multiple Myeloma	0.21
C0007102	Malignant tumor of colon	*C0024299*	Lymphoma	0.21
C0024299	Lymphoma	*C1306460*	Primary malignant neoplasm of lung	0.21
C0011847	Diabetes	*C0026764*	Multiple Myeloma	0.21
C0017638	Glioma	*C0024299*	Lymphoma	0.21
C0024299	Lymphoma	*C0684249*	Carcinoma of lung	0.21
C0023418	Leukemia	*C1832661*	ANOPHTHALMIA AND PULMONARY HYPOPLASIA	0.21
C0021051	Immunologic Deficiency Syndromes	*C0023418*	Leukemia	0.21
C0024121	Lung Neoplasms	*C0026764*	Multiple Myeloma	0.21
C0024299	Lymphoma	*C0242379*	Malignant neoplasm of lung	0.21
C0007131	Non-Small Cell Lung Carcinoma	*C0024299*	Lymphoma	0.21
C0024299	Lymphoma	*C0026769*	Multiple Sclerosis	0.21
C0024299	Lymphoma	*C0178874*	Tumor Progression	0.21
C0026764	Multiple Myeloma	*C0033578*	Prostatic Neoplasms	0.21
C0026764	Multiple Myeloma	*C0751606*	Adult Acute Lymphocytic Leukemia	0.20
C0009402	Colorectal Carcinoma	*C0026764*	Multiple Myeloma	0.20
C0026764	Multiple Myeloma	*C0598766*	Leukemogenesis	0.20
C0023418	Leukemia	*C0029408*	Degenerative polyarthritis	0.20
C0024299	Lymphoma	*C3539878*	Triple Negative Breast Neoplasms	0.20
C0023418	Leukemia	*C0345904*	Malignant neoplasm of liver	0.20
C0024299	Lymphoma	*C0302592*	Cervix carcinoma	0.20
C0024299	Lymphoma	*C4048328*	cervical cancer	0.20
C0023418	Leukemia	*C0026769*	Multiple Sclerosis	0.20
C0024299	Lymphoma	*C0699791*	Stomach Carcinoma	0.20
C0023418	Leukemia	*C0024305*	Lymphoma. Non-Hodgkin	0.20
C0024299	Lymphoma	*C4722085*	Malignant neoplasm of colon and/or rectum	0.20
C0024299	Lymphoma	*C4722518*	Triple-Negative Breast Carcinoma	0.20
C0004153	Atherosclerosis	*C0024299*	Lymphoma	0.20
C0026764	Multiple Myeloma	*C0596263*	Carcinogenesis	0.20
C0009324	Ulcerative Colitis	*C0024299*	Lymphoma	0.20
C0026764	Multiple Myeloma	*C0027627*	Neoplasm Metastasis	0.20
C0024299	Lymphoma	*C0024623*	Malignant neoplasm of stomach	0.20
C0003850	Arteriosclerosis	*C0024299*	Lymphoma	0.20
C0024299	Lymphoma	*C0600139*	Prostate carcinoma	0.20
C0024299	Lymphoma	*C1832661*	ANOPHTHALMIA AND PULMONARY HYPOPLASIA	0.20
C0021051	Immunologic Deficiency Syndromes	*C0026764*	Multiple Myeloma	0.20
C0024299	Lymphoma	*C0152013*	Adenocarcinoma of lung (disorder)	0.20
C0024299	Lymphoma	*C0376358*	Malignant neoplasm of prostate	0.20
C0024299	Lymphoma	*C0029408*	Degenerative polyarthritis	0.20
C0026764	Multiple Myeloma	*C1961102*	Precursor Cell Lymphoblastic Leukemia Lymphoma	0.20
C0026764	Multiple Myeloma	*C0678222*	Breast Carcinoma	0.19
C0019829	Hodgkin Disease	*C0023418*	Leukemia	0.19

C0009324	Ulcerative Colitis	*C0023418*	Leukemia	0.19
C0007847	Malignant tumor of cervix	*C0024299*	Lymphoma	0.19
C0024299	Lymphoma	*C0279626*	Squamous cell carcinoma of esophagus	0.19
C0002395	Alzheimer's Disease	*C0026764*	Multiple Myeloma	0.19
C0024299	Lymphoma	*C0033578*	Prostatic Neoplasms	0.19
C0024299	Lymphoma	*C0345904*	Malignant neoplasm of liver	0.19
C0011847	Diabetes	*C0024299*	Lymphoma	0.19
C0006142	Malignant neoplasm of breast	*C0026764*	Multiple Myeloma	0.19
C0024299	Lymphoma	*C0686619*	Secondary malignant neoplasm of lymph node	0.19
C0011849	Diabetes Mellitus	*C0024299*	Lymphoma	0.19
C0026764	Multiple Myeloma	*C1269955*	Tumor Cell Invasion	0.19
C0026764	Multiple Myeloma	*C1306459*	Primary malignant neoplasm	0.18
C0002395	Alzheimer's Disease	*C0024299*	Lymphoma	0.18
C0024299	Lymphoma	*C2239176*	Liver carcinoma	0.18
C0009402	Colorectal Carcinoma	*C0024299*	Lymphoma	0.18
C0024299	Lymphoma	*C0596263*	Carcinogenesis	0.18
C0024299	Lymphoma	*C0678222*	Breast Carcinoma	0.17
C0024299	Lymphoma	*C0027627*	Neoplasm Metastasis	0.17
C0006142	Malignant neoplasm of breast	*C0024299*	Lymphoma	0.17
C0024299	Lymphoma	*C1306459*	Primary malignant neoplasm	0.16
C0026764	Multiple Myeloma	*C4721532*	Lymphoma. Non-Hodgkin. Familial	0.16
C0024299	Lymphoma	*C1269955*	Tumor Cell Invasion	0.16
C0026764	Multiple Myeloma	*C0079772*	T-Cell Lymphoma	0.15
C0023418	Leukemia	*C4721532*	Lymphoma. Non-Hodgkin. Familial	0.15
C0023418	Leukemia	*C0079772*	T-Cell Lymphoma	0.14
